# A Real-Time Construction Safety Monitoring System for Hazardous Gas Integrating Wireless Sensor Network and Building Information Modeling Technologies

**DOI:** 10.3390/s18020436

**Published:** 2018-02-02

**Authors:** Weng-Fong Cheung, Tzu-Hsuan Lin, Yu-Cheng Lin

**Affiliations:** 1 Department of Civil Engineering, National Taipei University of Technology, Taipei 10608, Taiwan; weng-fong@hotmail.com; 2 Department of Civil Engineering, National Central University, Taoyuan 32001, Taiwan; cornetlin@gmail.com

**Keywords:** WSN, IoT, BIM, hazardous gas, safety management, job site, monitoring

## Abstract

In recent years, many studies have focused on the application of advanced technology as a way to improve management of construction safety management. A Wireless Sensor Network (WSN), one of the key technologies in Internet of Things (IoT) development, enables objects and devices to sense and communicate environmental conditions; Building Information Modeling (BIM), a revolutionary technology in construction, integrates database and geometry into a digital model which provides a visualized way in all construction lifecycle management. This paper integrates BIM and WSN into a unique system which enables the construction site to visually monitor the safety status via a spatial, colored interface and remove any hazardous gas automatically. Many wireless sensor nodes were placed on an underground construction site and to collect hazardous gas level and environmental condition (temperature and humidity) data, and in any region where an abnormal status is detected, the BIM model will alert the region and an alarm and ventilator on site will start automatically for warning and removing the hazard. The proposed system can greatly enhance the efficiency in construction safety management and provide an important reference information in rescue tasks. Finally, a case study demonstrates the applicability of the proposed system and the practical benefits, limitations, conclusions, and suggestions are summarized for further applications.

## 1. Introduction

International occupational safety statistics indicate that the construction industry has one of the highest industry accident rates [1]. Many construction sites are underground or in confined spaces, where the working envirement is not only harsh, but also potentially harmful gases may exist, and the accident statistics also indicate that many fatalities and injuries in confined spaces are due to exposure to the hazardous environment. A “confined space” as defined by the Occupational Safety and Health Administration (OSHA) [2] is as space large enough for an employee to enter fully and perform assigned work, but not designed for continuous occupancy by the employee, and has a limited or restricted means of entry or exit. These spaces may, but are not limited to, tanks, vessels, silos, storage bins, hoppers, vaults, pits, manholes, tunnel, equipment housings, ductwork, pipelines, etc.

According to the National Census of Fatal Occupational Injuries survey conducted by the U.S. Bureau of Labor Statistics in 2015, there were 4836 workers killed on construction sites due to illness and fatalities, 9% of injuries were due to the exposure to hazardous environment and 3% of injuries were caused by fire and explosions [3]. The results of the accident survey show that the major causes of the disaster usually include the unique nature of the industry, the mistaken behavior of a person, the harsh environment, and poor safety management [4]. These problems and situations can potentially be improved by advanced information and communications technologies. 

In recent years, many new technologies have brought great potential applications for safety management. Wireless Sensor Networks (WSNs) are one of the most key technologies of “IOT” applications. Many independently operating sensor nodes compose a network which can collect, store and process environmental conditions, without relying on any pre-existing infrastructure. With low cost, small size, low power requirements, different sensing capabilities and dynamic networking characteristics [5], WSNs are suitable for use in the non-infrastructure field or hardly maintained places such as hillsides, plains or viaducts and tunnels, where they can collect environmental information (such as temperature, light, vibration, etc.) and return it to a host computer for further control, management and analysis tasks.

Building information modeling (BIM) is an advanced technology in construction industry development, which integrates different kinds of construction information into a 3D digital model and can be applied in all stages of a project lifecycle, such as planning, design, construction, operation and maintenance (O & M) [6]. The US National Institute of Building Sciences (NIBS, Washington DC, USA) defines BIM as “a digital representation of physical and functional characteristics of a facility” [7]. In safety application concerns, the advanced construction safety management starts at the project design phase and remains throughout the whole life-cycle. Initial attempts to decrease occupational injury rates consider the safety aspects in the design phase and the development of manual safety processes in the execution phase. The application of BIM is currently experiencing rapid growth in construction operations, planning and management, as well as in safety management [8].

This study integrates BIM and WSN technologies to enhance the safety management of hazardous gas in an underground construction site, where the hazardous gas concentrations and environmental conditions (temperature & humidity) of different site regions were collected and integrated on the site BIM model. The corresponding model components will dynamically display all conditions collected from sensor nodes with changing colors and indicate the safety status, which enables the complete safety status to be mastered on an “active” BIM model. The provided information can be referred to the regular job site safety management and emergent rescue tasks. Additionally, the designed intelligent function enables the system to remove hazards automatically when detecting abnormal situations. Therefore, the purpose of the study are: (1) to propose the approach of construction site safety management by integrating WSN and BIM technologies ; (2) to develop BIM-WSN based hazardous gas monitoring modules and system; (3) discussion of the benefit and the limitation of the proposed system.

## 2. Related Works

With the fast increasing IoT applications in recent years, applications of WSNs also became widely spread, on the other hand, the spatial data and asset data of BIM can provide a framework for the organization and analysis of IoT data, that is meaningful to building operations and provide a basis for considering BIM as a potential component of IoT [9]. The categories of research relative to WSN and BIM applications in recent years mainly include: (1) localization and safety management, (2) facility and environment monitoring, (3) building & infrastructure monitoring, (4) resource management and optimization, (5) equipment automation and efficiency enhancement. Some representative studies were introduced as follows. 

### 2.1. Localization and Safety Management

Li et al. introduced an environment-aware beacon deployment algorithm designed by the authors to support a sequence-based localization schema for locating first responders and trapped occupants at building fire emergency scenes [10]. Naticchia et al. surveyed the feasibility of an infrastructureless real-time monitoring system to provide prompt support for inspectors in charge of health and safety management on construction sites [11]. Wu et al. analyzed the proactively preventive information requirement of struck-by-falling-object accidents and proposed an integrated information management model using a ZigBee RFID sensor network to fulfill the requirements [12]. Ding et al. presented a real-time safety early warning system to prevent accidents and improve safety management in underground construction, based on the IoT technology [13]. Ding et al. presented a multi-node harmful gases acquisition system based on wireless sensor network technology to solve the problem of harmful gases leaking in a dangerous petrochemical environment [14].

### 2.2. Facility and Environment Monitoring

Marzouk and Abdelaty utilized a Building Information Modeling (BIM) and global ranking system to monitor Indoor Environmental Quality (IEQ) by monitoring indoor temperatures and particulate matter (PM) levels. A rating system was also developed and showed the effectiveness in ranking maintenance actions globally [15]. Riaz et al. attempted to reduce Health and Safety (H & S) hazards and developed a monitoring system based on BIM and wireless sensor technology. Industry feedback on the prototype indicated that the proposed solution could facilitate intelligent monitoring of confined spaces [16]. Yang and Ergan found a decrease in accuracy and efficiency in facility maintenance mainly due to the lack of spatial context and equipment status monitoring. The findings showed that visualization-based interfaces generally improve the accuracy and efficiency of facility operators’ decisions in monitoring tasks [17]. Hsieh and Lu proposed a visualization system that integrated monitoring information systems, three-dimensional digital field models, interactive computer graphics, and visualization techniques for the visualization of monitoring data along with field models. The system enables users to reduce misinterpretation, improves communication efficiency, and facilitates efficient decisions [18].

### 2.3. Building & Infrastructure Monitoring

Chae et al. developed a bridge health monitoring system and tested it in place of forty-five sensors of five types among of the sensors used extensively in suspension bridges to prove the viability of wireless sensor network in actual implementation [19]. Liu et al. integrated RFID, GPS, GIS, PDA, and GPRS technologies to develop an automatic control and real-time monitoring system for dam material transport truck watering. It achieves not only automatic and high-accuracy truck-and-material-based watering process but also makes possible timely feedback for watering control and improvement, ultimately enhancing the compaction quality of dam materials and realizing better project performance [20]. 

### 2.4. Resource Management and Optimization

Zekavat et al. presented a wireless site-network concept consisting of information hubs enabled to automatically connect data sinks with sources supported by software agents and connected the work crew electronically to the project network while collecting automatically live “as-built” data [21]. Cho et al. developed USN hardware toolkits for hoists, which aims to automate the vertical material delivery by sensing the material information and routing it automatically to the right place. The gathered information from the sensors can also be used for monitoring the overall status [22].

### 2.5. Equipment Automation and Efficiency Enhancement

Shin et al. developed a seamlessly integrated information management framework that can provide logistics information to project stakeholders for their decision making. The pilot test of the framework developed in the research showed that it can improve time efficiency by about 32% compared to the traditional supply chain management [23].

The literature review indicates some researches about the applications of facility management and energy efficiency monitoring had begun to adopt BIM-sensor technologies since 2008 and further promoted the development of the “intelligent buildings”. In recent years, with the advanced development of sensor and wireless network technologies, the applications and researched issues are becoming more diversified and profound. In addition to facility management and energy efficiency monitoring, the other issues about personal safety, intelligentization and resource optimization were also extensively researched. Furthermore, due to the decreasing of sensor size and cost, the more quantities of sensors can be devoted to support the “big data” collection and provide detailed information for analysis, or the sensor can be set on a moving person or a creature which really observes the interaction between human and ambiance, thereby the further detail researches about the health and safety of humans can be obtained.

## 3. System Planning and Design

For developing the proposed system and meeting the requirement of implementation, the following processes were performed including: (1) user requirement analysis; (2) system framework design; (3) system development and testing; (4) evaluation and improvement. The detailed are described as follows.

### 3.1. Requirements Analysis of Users

To understand the current hazardous gas management and requirement in construction safety management, five experts with over two decades of experience in construction site and metro tunnel safety management were interviewed and provided the information about the types of detector commonly used on construction sites and their relative limitations, and problems encountered in practice. The collected opinions and suggestions will be referenced for the system development. The survey results show that the most common hazardous gases in construction include methane and hydrogen sulfide, which are usually emitted from the ground during excavating tasks, so the concentration has to be monitored and controlled, especially in underground sites or in confined spaces. In general, the staff uses handheld type gas detectors on construction sites, which are carried by the staff and detect any nearby hazardous atmosphere. The other type of gas detector is “fixed”, which was usually installed at a high risk or important location and transfers data to a host by wired lines. In practical terms, the usage of handheld detectors has a certain degree of risk, because the staff has to come in contact with the hazardous atmosphere during operation, while fixed type gas detectors may have increased maintenance costs for the erection or migration of the wire lines, especially when the working regions of a site are frequently moved.

To summarize, the relevant opinions and requirements about construction hazardous gas safety management include: (1) to enhance the safety of operator; (2) requirement for long-term and continuous data recording; (3) the site region monitored in the measuring task can be easily expanded; (4) to increase the convenience in measuring operations; (5) expandability of detectors for various hazards; (6) long-term data preservation for analysis and management; (7) to preserve the locational information accompanying the measurements; (8) automatic control and other intelligent functions.

### 3.2. System Planning

**Figure 1:**
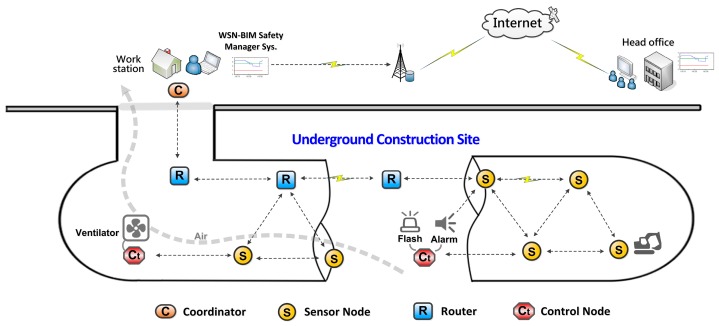
The concept of the hazardous gas detection system.

The system is planned to establish a WSN monitoring system by installation of sensor nodes in an underground construction site, to collect hazardous gas, temperature and humidity condition information. The collected data is then integrated into a BIM model and dynamically shows the safety status by colors according to the real detected conditions. Intelligent functions that respond to high concentrations of hazardous gases were also planned, which enable the system to automatically activate a flashing alarm and ventilator to warn workers and prevent from serious gas-hazard disasters. The concept of the planned system is shown in Figure 1.

### 3.3. System Analysis

**Figure 2:**
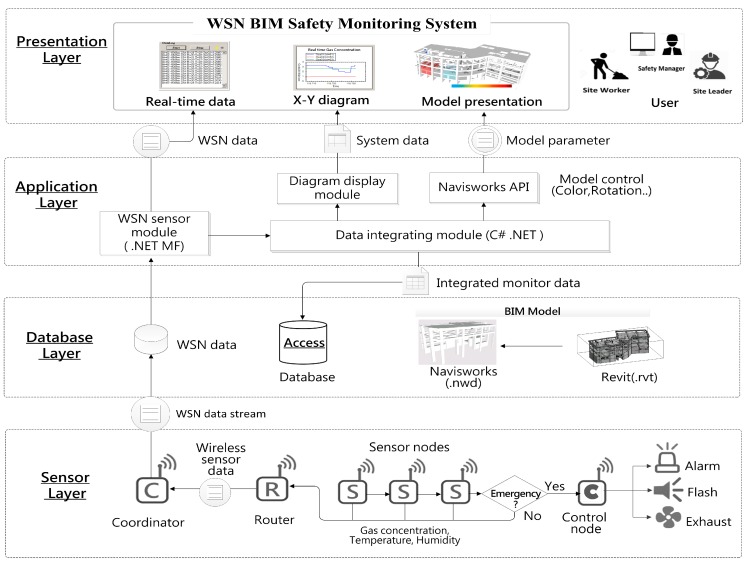
The multi-tier architecture and data flow of the system.

A multi-tier architecture was adopted for the design work and to distinguish the proposed system, which includes: (1) presentation layer; (2) application layer; (3) database layer and (4) sensor layer. Different attribute of entities were classified by layers and to clarify the relationships between the various subsystem and modules. Figure 2 demonstrates the multi-tier architecture and data flow analysis of the system. The descriptions and planning tasks of each layer are listed in Table 1.

## 4. System Development

Two different categories of subsystems, including WSN and BIM, were integrated into the main system in the development phase. The WSN system comprises different kinds of sensor nodes, where every node was an independently operating device which was programmed by .NET Micro Framework (.NET MF), and operated on a system-embedded chip. The nodes compose a WSN system which was responsible for detecting the environmental conditions, returning data and responding to emergencies. The other subsystem was established on a BIM model, which contains a digital model of the monitoring site and many components for displaying the spatial information and observed conditions. The model was built by Autodesk Revit (.rvt file) applications and then converted to a Navisworks (.nwd) file for reducing the file size and allowing control by a program. The model provides both location and safety information by visualization.

The overall system was integrated by the Microsoft Visual Studio 2013 and C# application. All the WSN data from different monitoring locations, including hazardous gas levels, temperature and humidity conditions, will be sent back by a relay of nodes and collected by the system. Then the components of the BIM model corresponding to the site monitoring location will be integrated with the returned WSN data and dynamically display colors indicating the hazardous gas and environmental conditions. The overall safety status of the whole site can thus be easily observed and realized on an active BIM model in real time.

**Figure 3:**
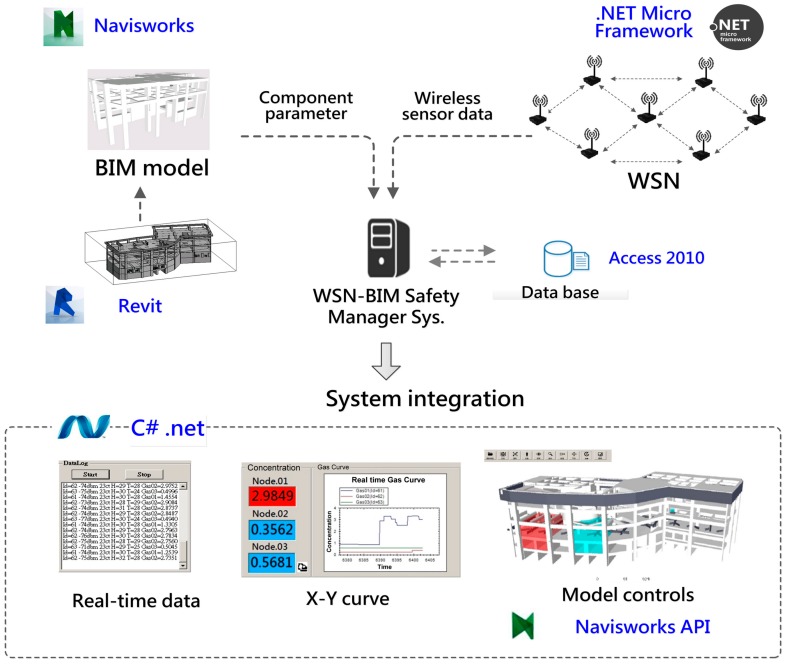
The system relationship and adopted applications.

To summarize, the tasks in the development phase include: (1) development of WSN sensor nodes, system and networking test; (2) establishment of a BIM model and modification; (3) integration of WSN data and the BIM model; (4) database creation and data processing; (5) system implementation and testing. The proposed items, adopted applications and functions are shown in Table 2, and the relations between subsystems and relative adopting applications are shown in Figure 3.

### 4.1. Network Topology and WSN Node Design

#### 4.1.1. Network Topology

**Figure 4:**
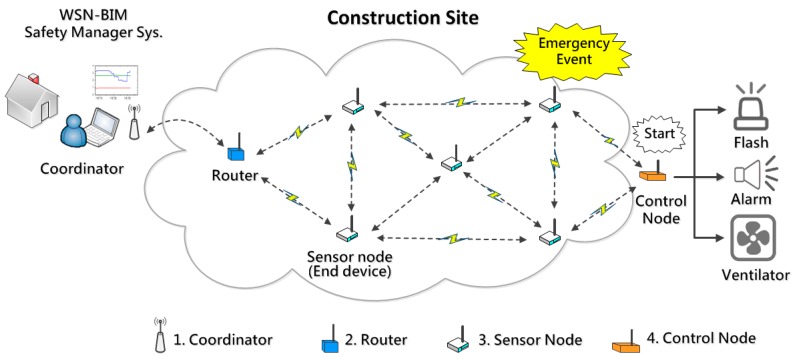
The mesh topology of the proposed WSN system.

Wireless network topologies typically are divided into “star”, “tree” and “mesh” layouts [24]. In a star topology, every node of the network is attached individually to a central node (host). The data is directly sent to the host without any relay, so the transmission distance is shorter and maybe limited. A tree topology network is a combination of star networks in a clustered arrangement, which can also possibly become disconnected if the head node of the cluster fails. In a mesh topology network, every node cooperates with the others for data transmission; the data can be sent over far distances by ad hoc node relays. The topology can also be automatically recovered by WSN self-connecting capabilities if any node fails. Considering the environment of construction sites is usually poor and harsh, and the sensor nodes may be damaged by high temperature, humidity or mud pollution, the system adopts a “mesh” type WSN topology, which is advantageous to provide longer communication distances and automatic network recovery capabilities. During the data communication process, when a node fails, the parent node will automatically find another node to connect to and resume the data communication, which is helpful in avoiding data loss and preserve system operation (Figure 4).

#### 4.1.2. The Developments of WSN Node

The wireless sensor node WSN system adopts the Microsoft “Gadgeteer” embedded system as the hardware framework, on which the programs and operational functions were developed by applying the Microsoft .NET Micro Framework (.NET MF). Each node is an independently operating device that forms a network using wireless Zigbee modules. A typical WSN system usually contains several types of nodes such as “Coordinators”, “Sensor nodes” and “Routers.” The “Coordinator” is connected to the computer and can be only one, which is responsible for launching the WSN network and acts as a “host” to collect the data from all the sensor nodes. The “Router”, without sensing capabilities, only relays the data from parental nodes to the next node, which extends the signal transmission distance and network coverage. The “Sensor nodes” (end devices) are equipped with hazardous gas (methane), temperature and humidity sensing components and are responsible for collecting and returning the detected data to the host. A “Control node” is also developed to enable the system to have intelligent early warning functions and actively prevent hazards. When any sensor node detects an abnormal situation, the control node will be notified to start a flash alarm and ventilator for warning humans to evacuate and exhausting the hazard. The types of developed WSN nodes in this study are listed and described in Table 3 and two characteristic types of WSN nodes (sensor node and control node) were introduced in details as follows.

##### Sensor Node Development

**Figure 5:**
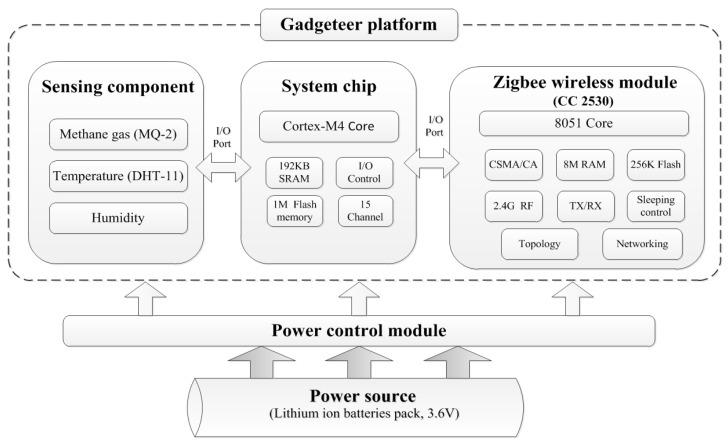
The hardware framework of sensor node.

The “sensor node” is the main device for detecting hazardous gas and environmental conditions. The module comprises the following parts (Figure 5):

(1) Gas sensor: a MQ-2sensing component [25] is used for detecting the methane gas concentration and transform it into a corresponding voltage for signal output.
(2) Temperature and humidity sensor modules: DHT-11 [26] sensor modules which can detect the ambient temperature and humidity and then transform them into digital signal outputs are used.
(3) System main board: Microsoft “Gadgeteer”, including an ARM Cortex-M4 core processor, was adopted as the hardware architecture of the embedded system.
(4) Wireless transmission module: the detected data was transmitted through the Zigbee CC2530 wireless module [27]. The networking type of the module is debugged as a “mesh” topology.
(5) Power supply module: composed by several 18650 lithium ion batteries and providing regular 3.6 V power for the operation of the sensor nodes.

**Figure 6:**
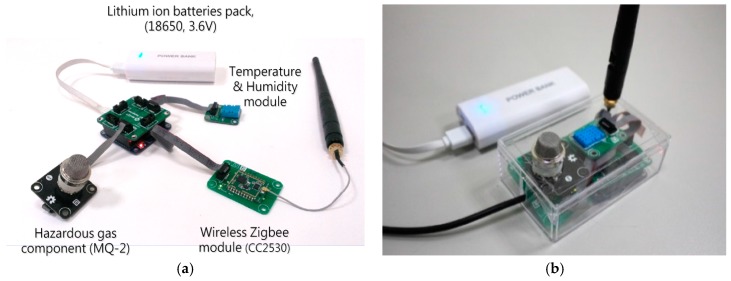
The developed sensor node (**a**) disassembled; (**b**) compacted.

**Figure 7:**
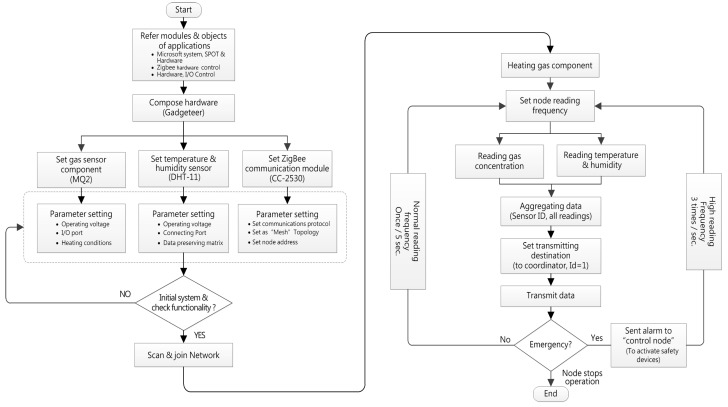
The flowchart of the sensor node program design.

The above sensor components were installed on the Gadgeteer mainboard and connected by cable lines. The main board has a stacked-up structure which provides several ports for different kinds of sensor component and functional modules to connect. The data detected by sensors was transformed and aggregated by the processor and then transmitted by the wireless module. The sensor node was intelligently designed to record data at a lower frequency (once/5 s) in usual operation for saving power, but once an abnormal hazard is detected, the system automatically increases the sampling frequency for exact recording (3 times/s), as the detailed records will be contributive to the follow-up investigation. Meanwhile, the sensor nodes will notice the control node to start the safety devices that avoids the accumulation of hazardous gas. The developed sensor node is shown in Figure 6 and the programming flowchart was shown in Figure 7. 

##### Control Node Development

**Figure 8:**
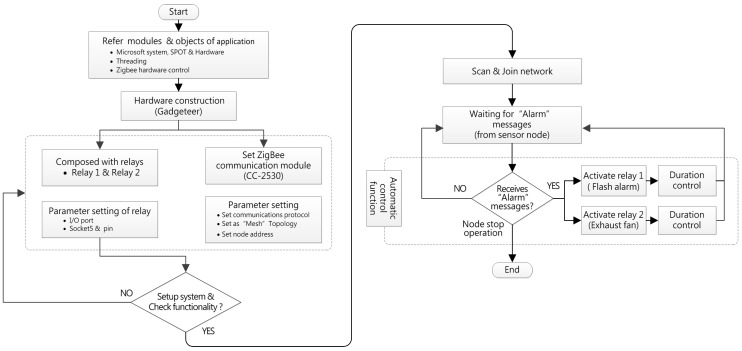
The flowchart of control node program design.

**Figure 9:**
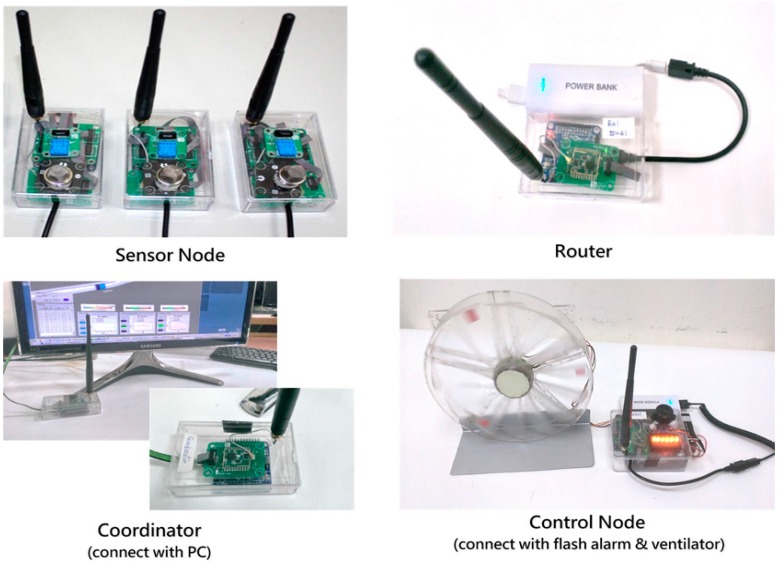
The types of all proposed WSN nodes.

The main function of the “control node” is to perform warning tasks and remove the hazards in an emergency situation. The node was designed to equip two set relays which connect to a flashing alarm and a ventilator. When any sensor node of the network system detects an abnormal gas concentration, the control node will be notified to start the flash alarm and ventilator, which warns humans to evacuate and removes the hazardous gas to preventing any gas accumulation and possible explosions. The programming flowchart of the control node is shown in Figure 8 and all of proposed WSN nodes are shown in Figure 9.

### 4.2. Establish and Control the BIM Model

The BIM model provides a visualized platform for monitoring and data integration of the gas concentration, temperature and humidity information detected by WSN nodes that is dynamically presented on the model through visualization of colored components. The gas concentration from high to low is displayed by a color gradient from blue to red and indicates the safety status; the temperature and humidity status, ranging from comfortable to dangerous is also shown by a green to red color gradient. Therefore, all the hazardous gas and environment situations were integrated into an existing BIM model, where both the information of detected conditions and spatial relations of monitored locations were obtained at the same time, so the real-time active model interface can dramatically improve the efficiency of safety management of a construction site.

#### 4.2.1. BIM Model Establishment

**Figure 10:**
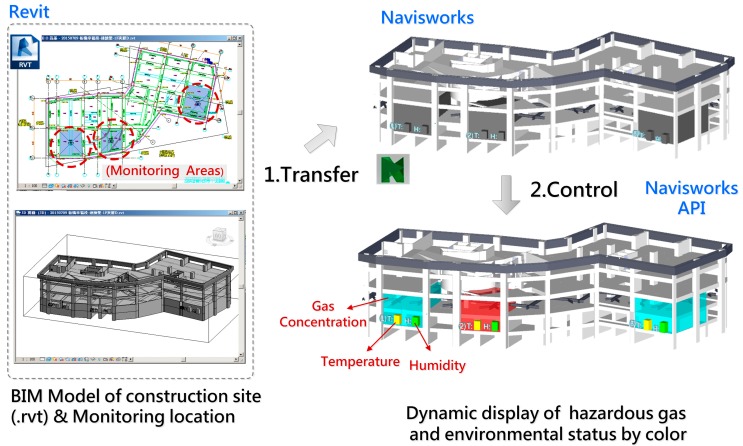
Construction of the BIM model and components.

The BIM model of the construction jobsite was built as an Autodesk Revit (.rvt file) and converted to a Navisworks (.nwd) file. The file still contains all the objects, properties, and geometries of the original model but with much reduced file size; the user can quickly view or operate the model using any version of the Navisworks product, even the free version. The BIM model structure should be appropriately modified and remove the external shield, that make sure the internal component can be observed when operating the model. Moreover, for the display of regional conditions, a “room” component corresponding to the monitored site location was created as a status indicator. For instance, the component representing gas conditions will actively change color according with the real-time gas concentration and indicates the safety status simultaneously; this mechanism is also applied to the monitoring of temperature and humidity conditions (Figure 10).

#### 4.2.2. BIM Model and Component Control

The Application Programming Interface (API) is a set of subroutine definitions, protocols, and tools for building application software. In general terms, it is a set of clearly defined methods of communication between various software components. API makes the development of computer programs much easier by providing all the building blocks [28]. In the system development, the Navisworks API was used to control the BIM model actions (e.g., moving, rotation, zoom and panning of the model) and change the component’s appearance (e.g., color or transparency). The functions were involved in the system interface which enables users to operate the model easily; or were referred to control the component color changes for indicating the regional conditions of construction site.

**Figure 11:**
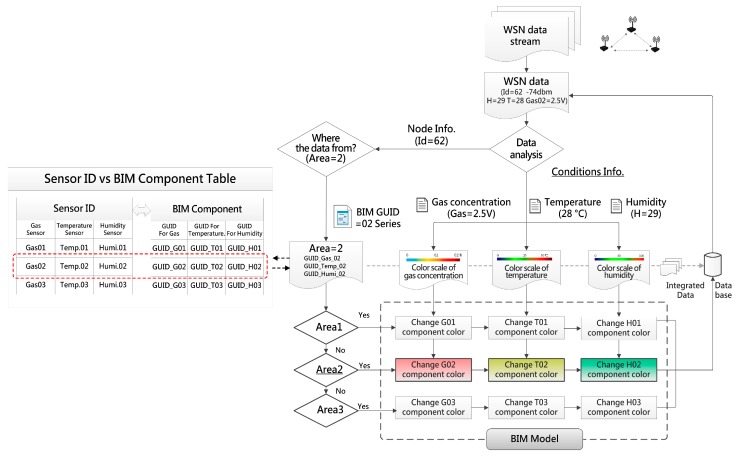
The integrating of WSN data and BIM component.

During the system operation, the system receives a WSN data stream through the coordinator, every piece of data contains two parts of information, including locational information and environmental conditions. The locational information will be referred to confirm which area/sensor the data comes from and to decide the correct display component on the model. The environmental information contains gas concentration, temperature and humidity data, the relevant data will be trimmed and to be a basis for acquiring the right color from the condition color scale. The color of components on the model will be actively changed and indicate the conditions according to every piece of the income data in real time. The integration process of WSN data and BIM component is shown in Figure 11.

### 4.3. System Integration

The overall system was integrated by Visual Studio 2013 and C# application which integrates the “WSN system”, “BIM model” and “database”. For improving the safety management efficiency, the system enhances the visualization of user interface, all the real-time measured gas concentration, temperature and humidity data were displayed on a data stream window, and on relative X-Y dynamic curve diagrams to show the varieties, additionally the data is turned into a dynamic-colored indicator to display the safety and environmental status of regional conditions.

**Figure 12:**
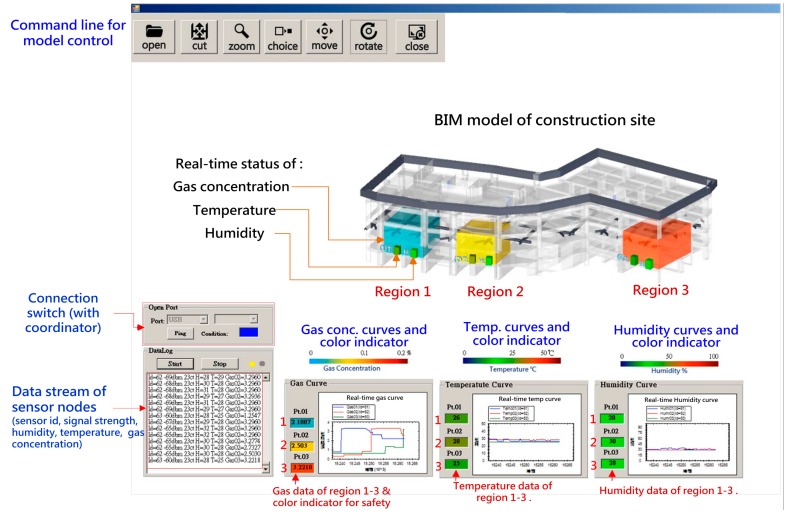
Screenshot of the integrated system.

After the system is started, the coordinator connecting with the computer first establishes a WSN network, then switch on the power of all WSN nodes sequentially (including the sensor nodes, routers, and control nodes), the nodes will join the network automatically and return data continually. The data from sensor nodes will be transferred by node relay from different measuring locations of the site and collected by the system through the coordinator. Every data point will be analyzed to check the represented location and the conditions of the job site. The measuring data including the date and time, address of node and signal strength, gas concentration, temperature and humidity conditions is preserved in a Microsoft Access file as a database for long-term management and analysis. A screenshot of the integrated system is shown in Figure 12. 

## 5. Experiments

### 5.1. Sensor Node Calibration

As the original measured gas data from the sensor node is a voltage value, it has to be further calibrated using standard gases and transformed into a meaningful gas concentration. The calibrated results will indicate the real gas concentration and that can be traced to international standards, and to realize if the concentration conforms to relevant regulations and does not affect human health. 

**Figure 13:**
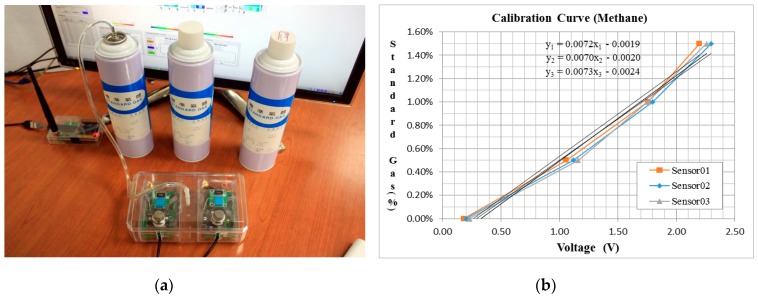
The calibration of sensor nodes, (**a**) Calibration by different concentration standard methane gases; (**b**) Relational curves of gas concentrations and measured voltages.

Methane gas standards of different concentrations were chosen as the calibration gas which includes 5000 PPM, 10,000 PPM (the American safety regulation level) and 15,000 PPM (the Japanese safety regulation level). In the calibration process, first, the sensor node was put into a sealed vessel which was then filled with the standard methane gas, then the measuring voltage will increase as the gas contacts the sensor. The voltage data will be sent out from the vessel by radio and received by the coordinator. When the value was stable, the voltage and gas concentration value were recorded. The process is repeated until all the various gas concentrations were calibrated. Finally, a relational curve between voltage and gas concentration can be regressed (Figure 13) and Equation (1) can be obtained:

*Y* = 0.0072*X* − 0.002 (1)

*Y: gas concentration (%), X: Voltage (V)*

The calibration result shows that each additional volt increase represents a 0.7% methane gas concentration increase. For example, for 1% concentration (10,000 PPM) of methane gas conforming to the US regulation, the corresponding voltage is 1.7 V, that means when the sensor node detects a voltage value that exceeds 1.7 V, it may exceed the regulation control value and cause harm of human health.

### 5.2. Node Performance Test

There are many methods to evaluate the performance of sensor nodes such as Received Signal Strength Indication (RSSI), Packet Delivery Rate (PDR) or Packet Loss Rate (PLR), etc. RSSI is a measurement of the power present in a received radio signal, which is a negative value in dBm (decibel-milliwatt) units; a greater value means a better transmission quality. In general, an RSSI value between −50~0 dBm means the signal strength is great; less than −80 means the signal is attenuated, and it’s completely disconnected if the signal is less than −100 dBm. The PDR is the ratio of the number of packets successfully received by a destination node to the packets that have been sent. The PLR is the ratio of the number lost packets to the sent packets, which is the reciprocal of the PDR. The PDR calculation is shown as Equation (2):

(2) $$\mathrm{PDR}\;\left(\text{\%}\right)=\frac{P_{Received}\times 100}{\sum_{i=1}^{n}P_{Generated}\;}$$

*P_Received_*: packets number that is successfully received by the destination node, *P_Generated_*: packets that have been sent, *n*: number of sensor nodes.

**Figure 14:**
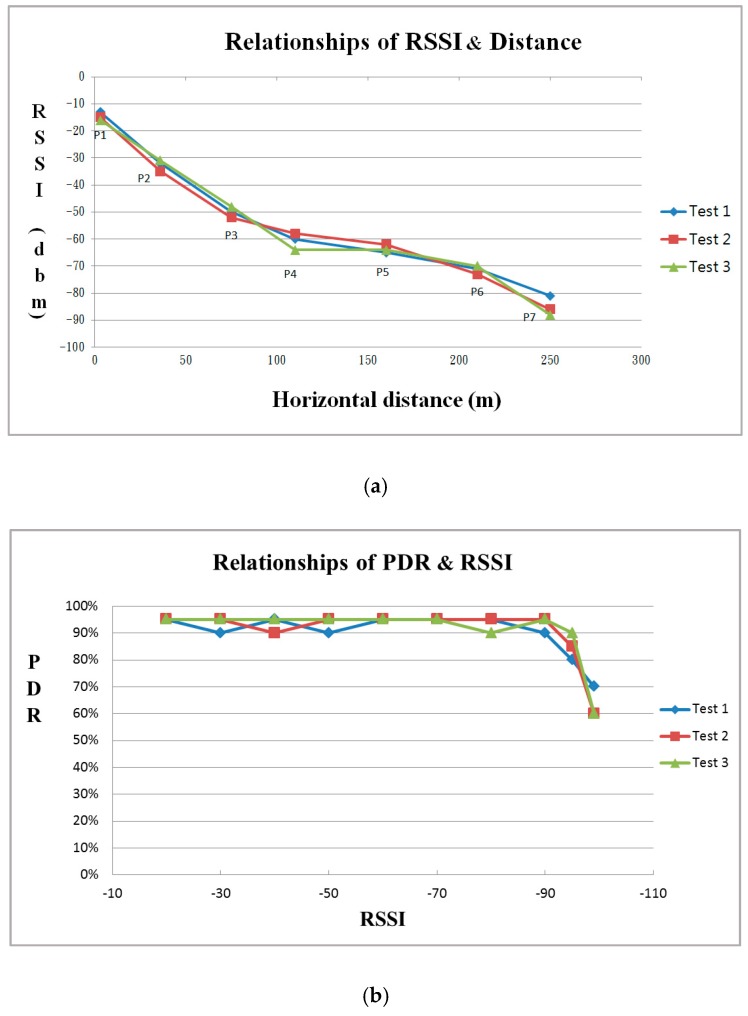
The relational curve of (**a**) RSSI and distance; (**b**) PDR and RSSI.

To test the communication performance of sensor nodes and determine the relations about RSSI and PDR, a sensor node was adopted to be the signal transmitter and was designed to send a data packet in one second. The receiving node connected with a notebook was used to calculate the number of received packets every 20 s. The transmitter was moved horizontally from the receiver away in regular intervals until the data cannot be received. The test result displays that when the moving distance is close to 250 m, the signal strength becomes seriously attenuated and the RSSI is decreased to about −95 dBm, that means if the separation distance is more than 250 m between two sensors, the data maybe lost or cannot be communicated (Figure 14).

## 6. Case Study

### 6.1. Test Processes

An underground construction job site of the MRT tunnel in northern Taiwan was chosen as the experimental site of the case study. The tunnel diameter is 5.6 m and 528 m in length. The site workers were doing the rail track installation work, and the machine operation and the cutting and welding operations were likely to produce harmful gases, the working environment was polluted and needed to be monitored for controlling the hazardous gas concentration.

**Figure 15:**
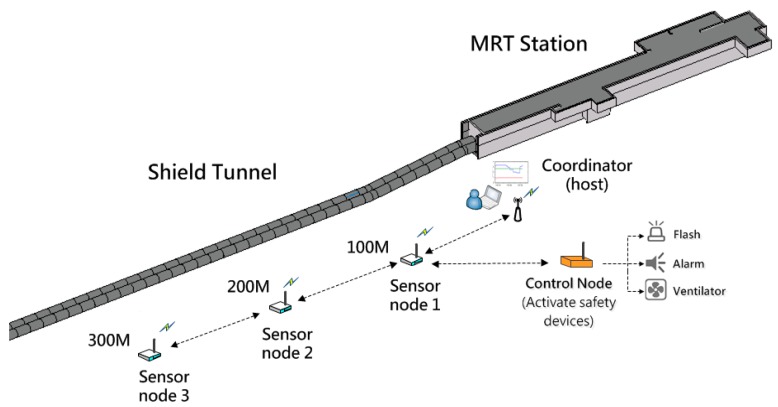
The layout of the WSN experiment in tunnel.

The experiment is designed to monitor 300 m of tunnel distance. The sensor nodes were placed every 100 m and three monitoring points were installed. The coordinator linked with a notebook (host) was set up in the station which was near the tunnel entrance. A BIM model of the tunnel construction site and the MRT station was established and set up in the monitoring system. Three annular components were also embedded in the tunnel model for indicating the conditions (gas concentration, temperature and humidity), so in total nine components were adopted to indicate the conditions of three monitoring regions. Additionally, a control node was set up in a location near the coordinator which is connected to a flash alarm and a ventilator. When any hazardous gas was detected within the range, the control node will automatically activate the flash alarm and ventilator for warning humans to evacuate and exhausting the hazardous gas. The layout of the experiment is shown in Figure 15.

The experiment in tunnel includes three testing tasks: (1) the performance of the system in gas detection; (2) the signal transmission and attenuation test; (3) the node interference test. The test description of each item is given in Table 4:

#### 6.1.1. Performance Test in Gas Detection

**Figure 16:**
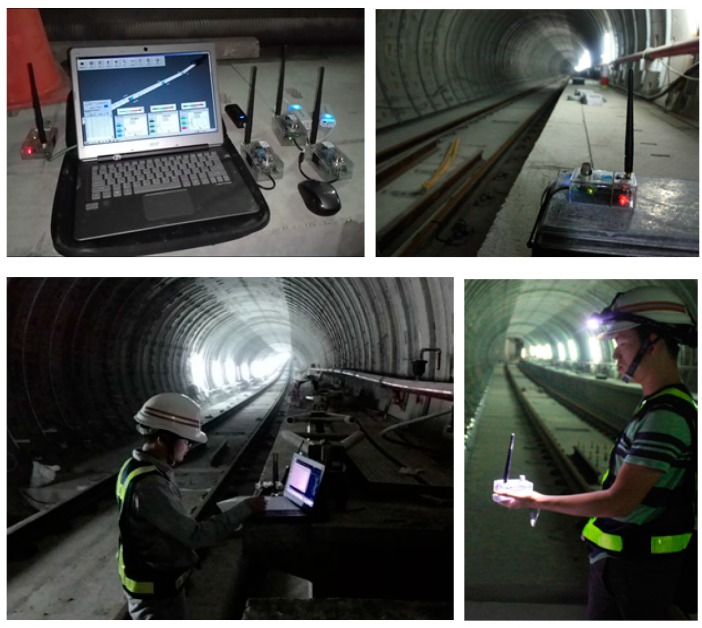
The WSN experiment in tunnel.

This test is to simulate the occurrence of hazardous gas and test the performance of the system. First a coordinator linked with a notebook was set up as the host (the system including the construction model has been set up in the notebook in advance). The coordinator launches a network when the system starts, and the sensor nodes, and the control node then auto-join the network in sequence after switching on the power. Later, the staff carries the sensor nodes and moves forward into the tunnel, the staff reports the reached position in every 20 m by radio, and then the staff on the host side record the received signal strength and departure distance. The staff sets up three sensor nodes respectively at the positions of 100, 200 and 300 m, and after all the sensor nodes have been set up, the staff simulates the occurrence of hazardous gas by emitting a combustible gas from a lighter and tests whether the system can immediately detect the emergency event (Figure 16).

**Figure 17:**
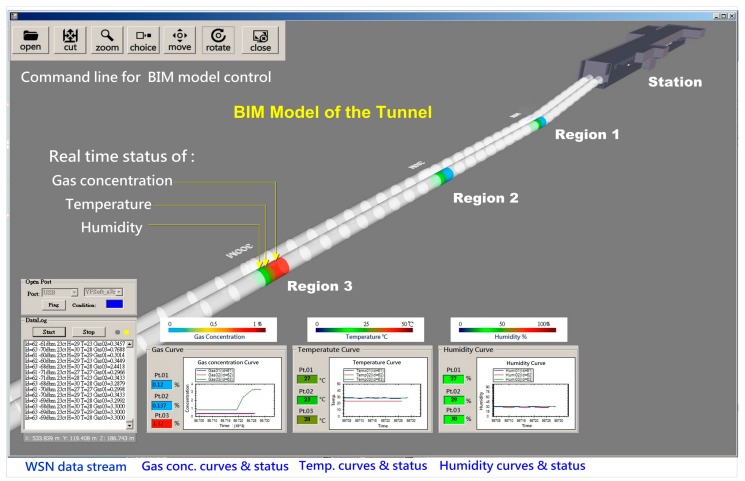
The screenshot of the WSN-BIM safety management system (test in tunnel). (Abnormal gas concentration was detected at region 3 and alerted by red color).

The experimental result displays that even in the position of 300 m, the system can immediately react to the hazardous gas occurrence in 1–2 s and alert the abnormal location on the system BIM model by the red color of the regional component. Simultaneously, the control node starts the flash alarm and the ventilator automatically, which warns the humans to evacuate and eliminates the risk of gas accumulation. The test result displays the system can perform the detection and control tasks well in the tunnel construction job site (Figure 17). 

#### 6.1.2. Signal Transmission Test

**Figure 18:**
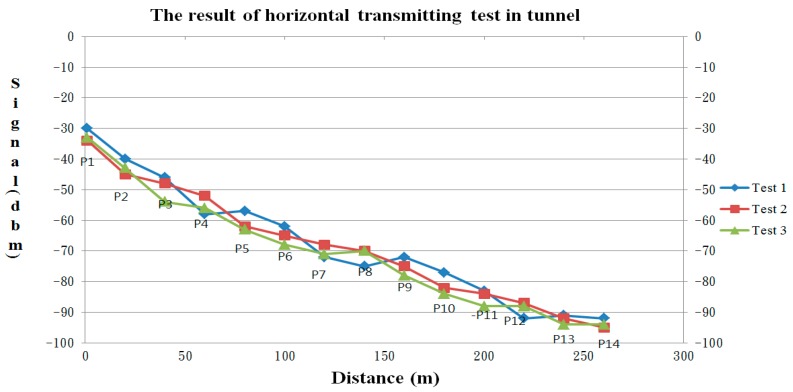
The WSN performance curve in the tunnel test.

After the above test has finished, the staff carries the sensor node to move around the tunnel continuously and test the transmission distance limitations. The staff moves and reports the position every 20 m and the respective signal strength was recorded. The test result displayed that the transmission distance in the tunnel can reach up to 250 m and the signal attenuation is about 0.2 to 0.3 dBm/m. Additionally, if a router was set up between two nodes, the transmission distance can be extended up to 450 m (Figure 18).

#### 6.1.3. Interference Test of Sensor Node

**Figure 19:**
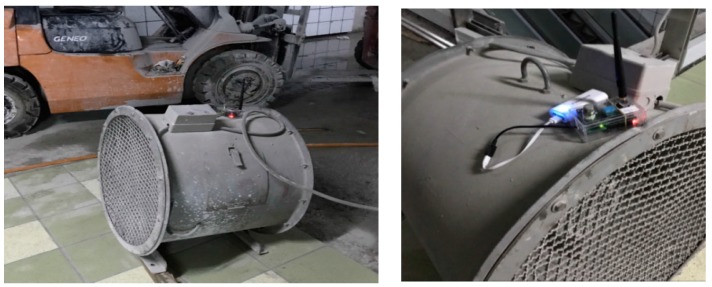
The interference test of sensor node.

This test is to observe whether the signal of sensor node will suffer interference from construction equipment such as high-speed ventilator or a running engine on a lift truck. An operating sensor node transmitting signal was put on the operating device; the strength and stability of the signal was then observed and compared with the original values. The test result displayed the signal was not significantly affected by operating equipment (Figure 19). 

### 6.2. Evaluation and Discussion

The case study demonstrated the applicability of the proposed system in underground construction sites; the function, transmission and stability of the system were tested and observed to understand the system performance. In general, the most popular gas detector on construction sites is “handheld” type due to its lower cost and convenience. Some important tasks adopt “fixed” type detectors for the purposes of continuous and important data collection, such as monitoring the position of tunnel boring machines during excavation. The WSN is a new developed technology which allows high potential applications and development in safety management.

With respect of the operation, the operation of a handheld detector has less safety because the user has to approach the hazardous gas atmosphere for checking or re-checking tasks; in regard to data collection the handheld detector requires manual labor, so the detection frequency is usually low and the data are mostly fragmental. WSN and fixed type detectors can perform long-term detection and monitor several locations at the same time; the data can also be preserved in a database for analysis and management.

In this study, the WSN uses either individual batteries pack or an external power supply (such as a 3.6 V transformer) as power source if the external power is not available or the node has to be set on a moving object that needs an individual power source. The test result indicate that a power module with six 18650 lithium ion batteries could support the sensor node operation for 5–6 days; a longer operation will need more power supply, so the duration of the power supply is an important issue of concern in an independent WSN system.

In the cost analysis, the price of each WSN sensor node is about 80 to 100 U.S. dollars (USD), and the detection at least needs both a sensor node and a coordinator, so the cost is about 200 USD for single point detection (without considering the cost of a notebook). If it’s necessary to increase the number of detection points, the cost will increase by 100 USD per node. Comparing with the handheld detectors (which cost from 200 to 400 USD/ea.) and the fixed type detector (400 to 700 USD/single point), if there are four locations need to be monitored, the cost of handheld detectors will be 800 USD (200 USD/ea.), the fixed type requires 2000 USD (400 USD/ea. plus any additional wiring cost) and the cost using the proposed WSN system is 500 USD (four sensor nodes and a coordinator), so the cost of the WSN system is 40% to 75% lower than the handheld and fixed type detectors.

Regarding the maintenance, the dimensions of the handheld detector and WSN devices are relatively compact and low unit price, which is easier for maintenance or replacement; the fixed type detectors are linked with wire lines, so if the detection position needs to be moved or increased, the wire lines have to be re-installed. The environment of construction sites is usually harsh and the work region is frequently changed, which increases the difficulties and cost of maintenance.

To consider the requirement of expandability and intelligent functions, the wireless or fixed type detectors are designed by combination of sensors and networks, which enables the detector to change sensing components separately and operate with the original network backbone. The handheld type detector offers the convenience of usage, the design is relatively regulative and the function is hard to expand. Furthermore, the proposed WSN gas detection system was designed by combining different sensor components and functional modules, which enable not only different sensing capabilities but also control capabilities. The sensor node could read data at a low frequency in usual operation for saving power, and when any of the sensor nodes detects an abnormal situation, the nodes will increase the data reading frequency, and a control node is notified to start the safety equipment operation and remove the hazards automatically. In particular, the WSN-integrated BIM model provides a visual and active platform for monitoring, the risk status of different site regions can be efficiently mastered by a colored and spatial display interface, and the real time safety status of different regions also provides important reference information for evacuation or rescue in emergency events.

## 7. Conclusions and Suggestions

### 7.1. Conclusions

1. Considering the characteristics of construction job sites, the network of “mesh” type topology within multi-hop and network auto-recovery capabilities is suitable for monitoring tasks in construction sites; “star” and “tree” type topologies networks may be limited by their shorter transmission distance and the communication may be interrupted by node failure.
2. The WSN provides a remote way for monitoring and controlling hazards which eliminates the risk of human exposure to hazardous environments and enhances the human safety in monitoring tasks.
3. Within the real-time safety status and spatial information displayed on the system model, the rescuer can determine the exact location of any event in advance, which is very important and helpful for rescue tasks in an emergency situation.
4. The designed intelligent function enables the system to automatically actuate the ventilation and related safety devices when an abnormal situation is detected, which can decrease the hazardous gas accumulation, and prevent possible explosions and human injuries.
5. The proposed system provides multi-condition monitoring information of several regions, which is helpful to judge the risk priorities of different regions (such as both high gas concentration and temperature conditions which are more dangerous than only one condition), and the information is useful for human evacuation and decision making.
6. Compared with the “fixed” type monitoring system, WSNs not only can be quickly implemented and are free from wire maintenance, but the measuring locations can be changed or increased in number as demanded at any moment, the system is maneuverable and flexible, which meets the monitoring requirements of the construction environment.

### 7.2. Suggestions

1. As the Navisworks application currently does not provide specific components for demonstration of the sensing status, the “room” component adopted to display the safety status in this study which may suffer from the hollow phenomenon when cutting the model. If the application can provide dedicated components for demonstrating the sensing conditions, that will be helpful to improve the display of monitoring tasks.
2. The gas components show slightly different values in detection even with the same specification, which leads some degree of error in the measurements. It is recommended to add a calibration function to the system for each component, which will be helpful to improve the measurement accuracy.
3. The power supply and the network lifetime are still the main limitations of the WSN system, especially the gas detection component has to be pre-heated before operation which will consume more power. It is suggested to increase the power supply capacity or introduce an external power supply.
4. It is suggested to further increase the intelligent functions of system, for instance, the system may automatically derive an algorithm of the best evacuation route in fire emergency scenes by using the data returned by the WSN and the BIM spatial information, or estimate the influence on human health of low-dose hazardous gas exposures from long-term observed information.

## Figures and Tables

**Table 1:** The description and task of designed layers.

Layer	Description	Task
1. Presentation Layer	Real-time data collection and presentation. An “active” BIM model for visualization of safety management. Decision of the representation way of different areas and conditions.	To design a visualized system interface for efficient system management. To establish an active data and curve displays of real time data from sensors. To establish a dynamic color displayed BIM model platform.
2. Application Layer	WSN nodes program & function design (embedded system). BIM model establishment and transform. Integration of WSN, model and detecting data in the system.	To decide the appropriate applications and methods for the achievement of desired system function. To arrange and integrate the input and output between applications including sensor, communication, model and system.
3. Database Layer	Database design for preserving sensor and system data. Management and inquiry of measuring data and history. Dimensional and conditional information treatment by BIM model.	To build the database for system data preservation and management. To establish site BIM model including site spatial and locational information. To design the components for displaying the monitoring conditions.
4. Sensor Layer	Wireless sensor work design for the underground construction site. Sensor node development includes: (a) Sensor node. (b) Router. (c) Control node. (d) Coordinator. Intelligent function design of the nodes.	To design and develop the wireless nodes of different purposes. To build Zigbee wireless sensor networks system and test. To collect the hazardous gas, temperature, and humidity information for each location of the site. To design the function of WSN nodes including automatically removing hazards and changing the data recording frequency.

**Table 2:** The proposed items and system functions.

Item	Application /Software	Function
1. Development of WSN nodes and the network system	.NET Micro Framework (.NET MF)	Sensor nodes: comprise gas, temperature, humidity components and Zigbee wireless modules to detect hazardous environmental gases, temperature, humidity conditions and return data. Coordinator: to collect the WSN returned data and transfer to a computer. Control node: to receive commands from the sensor nodes, and start the flash alarm and ventilation in an emergency. Router: to extend the WSN transmission distance and coverage.
2. Building of the BIM model and processing	Autodesk Revit Autodesk Navisworks Navisworks API	To build and modify the construction BIM model by Revit(.rvt) Convert to Navisworks (.nwd) format file which reduces the file volume. To control the actions of BIM and appearance color of components by Navisworks API.
3. Data processing	NET Framework, C# Access Excel	To receive and process the received data stream and transfer to the database and Excel file. Dynamic display of gas concentration and environmental information by X-Y curve diagram.
4. Integration of BIM and WSN information	NET Framework, C# Navisworks API	BIM model control (open, rotate, pan, zoom, etc.). To integrate gas concentration, temperature, and humidity information. To control the color changes of components and warn about abnormal regions.
5. Hazardous gas management information system	NET Framework, C# Access 2013	Storage and management of system data. Inquiry of historical information. Registration and management of node information.

**Table 3:** The proposed WSN nodes.

Name	Function
1. Coordinator	Responsible for launching and coordinating the WSN network. To be a host and collect information from all sensor nodes (into the computer).
2. Sensor node	To collect the hazardous gas, temperature and humidity information from different regions of the underground construction site. To notify the control node to start safety devices and remove hazardous gases. To record the detailed data of the duration of emergency.
3. Router	To relay the WSN data transmission from the parental node. To extend transmission distance and network coverage.
4. Control node	Connection with flash, alarm, and ventilator, activated in emergency situation and to remove the hazards.

**Table 4:** The experiment item in tunnel.

Items	Description
1. System performance	To set up a WSN network of 300 m coverage (100 m per node) for hazardous gas monitoring in the tunnel. To simulate the occurrence of hazardous gas and test the reaction of the system. To test the intelligent functions of the system (automatically start the flash alarm and ventilator).
2. Transmission distance test	To move sensor node horizontally, record the signal strength every 20 m until the signal disappears.
3. Router test	To test the signal transmission distance extended by relying with a router.
4. System interference	To set the sensor node on an operating machine (such as a high speed ventilator) and observe the signal interference.
